# How Do Psychological Cognition and Institutional Environment Affect the Unsafe Behavior of Construction Workers?—Research on fsQCA Method

**DOI:** 10.3389/fpsyg.2022.875348

**Published:** 2022-06-03

**Authors:** Beifei Yuan, Shuitai Xu, Li Chen, Muqing Niu

**Affiliations:** ^1^School of Economics and Management, Jiangxi University of Science and Technology, Ganzhou, China; ^2^First Affiliated Hospital, Henan University of Science and Technology, Luoyang, China

**Keywords:** unsafe behavior, safety performance, configuration perspective, social cognitive theory, fuzzy-set qualitative comparative analysis

## Abstract

The frequent occurrence of safety accidents is a global problem, and unsafe behavior is the main cause of accidents, which has been unanimously recognized by academia and industry. However, the previous research on unsafe behavior focused on analyzing the linear effects of variables on the results, and it was difficult to systematically analyze the complex mechanism of the results generated by the coupling of each variable. The problem of how to avoid unsafe behavior of construction workers has not been effectively solved. Based on the configuration perspective, on-site observation is organized, 164 construction workers are taken as case samples, the traditional regression analysis method is abandoned, and the fuzzy set qualitative comparative analysis method is used to integrate the theoretical framework of social cognition. From the perspective of psychological cognition and institutional environment, this paper discusses the differential matching of construction workers’ safety attitude, safety motivation, institutional control, safety training, and safety climate, and exploring the causal complex mechanisms that improve unsafe behavior among construction workers. The results show that: (1) The unsafe behavior of construction workers is the result of multiple factors. A single influencing factor does not constitute a necessary condition for the unsafe behavior of construction workers; (2) the path leading to the unsafe behavior phenomenon is not unique. Therefore, the high and unsafe behavior configuration of construction workers is summarized as “psychological cognition scarcity type,” “institutional environment scarcity type,” and “attitude-climate scarcity type”; (3) compared with “psychological cognitive scarcity type” and “institutional environment scarcity type,” “attitude-climate scarcity type” is more likely to cause unsafe behavior of construction workers; (4) a lower level of safety attitude or safety climate is more likely to cause high and unsafe behavior of construction workers; and (5) the non-high and unsafe behavior driving mechanism for construction workers is “comprehensive,” and there is an asymmetric relationship with the driving mechanism of the unsafe behavior of high construction workers. The research conclusions of this paper can help to broaden the theoretical framework of social cognition and provide new ideas and methods for how to improve unsafe behavior.

## Introduction

**Figure 1:**
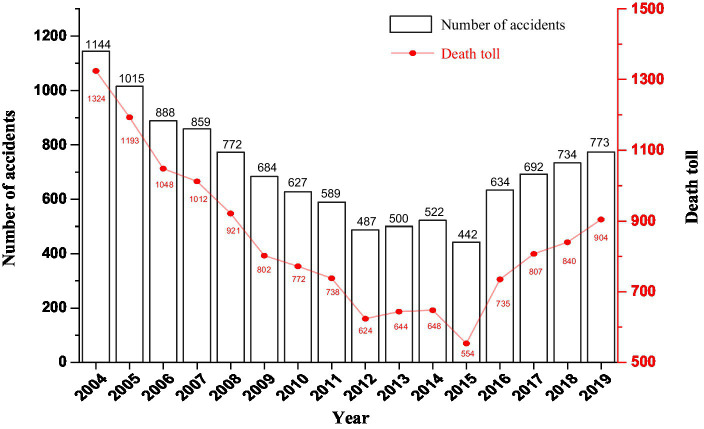
Distribution by number of Safety accident in construction from 2004 to 2019.

As a pillar industry in the world, the construction industry is one of the highest security risks in the world (Zaira and Hadikusumoa, 2017; Guo et al., 2021), and construction safety accidents cause construction worker casualties, heavy property losses, and serious negative social impacts (Feng et al., 2015). In China, although safer management safety intervention and technical safety intervention have been used in construction in recent years, the incidence of construction safety accidents is still high due to the large high-altitude operation and large personnel mobility. According to the results of the accident investigation report of the Ministry of Housing and Urban–Rural Development of the People’s Republic of China (Safety Production Management Committee), from 2004 to 2019, there were 11,362 safety accidents in construction and a total of 13,566 deaths (as shown in Figure 1). Therefore, the safety management improvement of the construction industry is facing huge challenges.

According to the existing safety accident investigation and the Domino theory (Heinrich, 1931), the direct cause of the safety accident can be classified: the unsafe behavior of construction workers and the unsafe state of objects (Choudhry, 2014; Fu et al., 2020). According to the analysis of the accidents in China’s construction industry, about 80% of the safety accidents were caused by the unsafe behaviors of the construction personnel, while the unsafe accidents caused by the unsafe conditions accounted for only 10% (Choi and Lee, 2018), indicating that the unsafe behavior of the construction workers is the main cause of the accidents. Based on the realistic point of view, considering the accident, construction workers often show inattention, lazy attitude, and at a loss in the face of the accident, showing that the people’s safety behavior management has omissions. Therefore, it is necessary to intervene and regulate the unsafe behaviors of construction personnel (Dekker, 2002), which is crucial to improve the construction safety management performance. Clarify the influence mechanism of construction worker unsafe behavior is an effective way to reduce safety accidents (Suto, 2009), analyze the factors that cause unsafe behaviors, clarify the complex relationship between construction factors, and propose solutions and interventions are the top priority of safety management performance (Yang et al., 2021).

The mechanism of unsafe behavior of construction workers is the focus of academic research (Choudhry, 2014; Guo et al., 2016a; Ding et al., 2018; Fang et al., 2018). Scholars have conducted research on the potential influencing factors of individual unsafe behaviors, including the micro-individual level and the macro-level institutional environment level, etc., and have obtained relatively rich research results. From the micro-individual level, construction workers’ safety attitude (Shin et al., 2014) and safety motivation (Panuwatwanich et al., 2017) are important factors affecting the unsafe behavior of construction workers. Previous studies have explored the influence mechanisms of personal emotion (Ju et al., 2016), safety cognition (Goh and Binte Sa’adon, 2015), autonomous motivation (Rigby and Ryan, 2018), work experience (Alizadeh et al., 2015), and individual characteristics on unsafe behaviors (Yang and Byung-Seok, 2014; Fang et al., 2015). It is believed that the construction site is different from other work situations, and the sudden safety accident is special, which is often caused by management defects and illegal behaviors at the same time (Reason, 1995). The negative impact of unsafe behavior is not immediately manifest, while individuals can get immediate benefits through violations, such as saving time and reducing the workload (Zohar and Luria, 2005). The negative effects of unsafe behaviors are not immediately manifested, but individuals can obtain immediate benefits through violations, such as saving time and reducing workload (Zohar and Luria, 2005). Interventions for unsafe behavior should follow a logic of appropriateness rather than a logic of consequences (March, 2010). That is to say, we should focus on realizing the self-efficacy perception of construction workers, enhance personal identification with roles and jobs, and pursue meaningful goals to achieve compliance with safe behaviors.

However, these studies ignore the key role of the macro-institutional environment level. In fact, construction workers are embedded in macro-institutional environments, such as enterprise, society and law, and construction enterprises provide workers with a workplace. Therefore, we began to investigate the influence of macro-institutional environmental factors, such as safety climate (Liao et al., 2014; Guo et al., 2016a), safety training, institutional supervision (Fang, 2006), safety incentive (Fam et al., 2017), social norms (Sampson et al., 2014), leadership commitment (Al-Refaie, 2013), and safety culture (Zhang et al., 2020a) on workers’ unsafe behavior. It is pointed out that the perception of workers in the institutional environment and the resources and support they can get during the construction process will lead to differences in individual behavior. However, human behavior is the result of the interaction between the environment and the individual (Lewin, 1976), and most of these studies focus on the influence of a single factor on the unsafe behavior of workers, ignoring the interaction between the micro-individual and the macro-institutional environment. With the deepening of research, from the perspective of cognitive psychology, people gradually realize that human behavior is mainly dominated by internal psychological activities, and psychological adjustment will be restricted by the external institutional environment. Therefore, it is necessary to explore the cognitive mechanism of construction workers to better improve the unsafe behavior of workers. Emerging research focuses on the impact of psychological cognition on worker unsafe behavior from the perspective of institutional environment interaction. Some studies have pointed out that the interaction between project managers and colleagues will affect unsafe behavior (Zhang et al., 2019), especially if managers ignore safety issues, which will have a negative impact on the safety awareness of team workers (Chang et al., 2019). Interaction between management and grassroots needs to be considered, and unsafe behavior of construction workers during construction may be corrected under social groups (Choudhry and Fang, 2008). For example, social groups often influence the unsafe behavior of construction workers through safety training and reward and punishment mechanisms. The social cognitive process that combines institutional environmental factors with psychological cognitive factors is complex, dynamic, and non-linear and needs to be identified with appropriate methods (Chang and Mosleh, 2007; Fang et al., 2016).

However, some studies have a clear methodological and theoretical mismatch, considering each research element as a whole. In terms of research methods, however, it only focuses on the impact of a single factor on workers’ unsafe behavior (Khosravi et al., 2014), which seriously restricts the strength of the explanation of workers’ unsafe behavior under the synergy of the individual and institutional environment as a whole. Fortunately, Qualitative Comparative Analysis (QCA) adopts a configuration perspective to effectively deal with causal relationships, such as multiple concurrency, and can fully explore the impact mechanism of the coordination and linkage of multiple levels of Causal conditions on unsafe behavior (Meyer et al., 1993). Therefore, Qualitative Comparative Analysis can be used to analyze the interactive influence of psychological cognition and environmental system on construction workers’ unsafe behaviors. It can avoid the deficiency of analyzing the “net effect” of single factor in regression analysis, and is more in line with the reality, making the analysis results more convincing.

Social Cognitive Theory (SCT) provides a systematic analytical framework for the study of individual behavior. An integrated theoretical framework is established from the two levels of personal psychological cognition and institutional environment, which can comprehensively consider multiple factors that affect individual behavior (Bandura, 2001). The generation of unsafe behavior of construction workers is a complex system, and the analytical framework provided by social cognitive theory can better cover the influencing factors of unsafe behavior of construction workers. Based on this, this study studies the configuration effects of different combinations of construction workers from five aspects: safety attitude, safety motivation, institutional control, safety climate, and safety training. In addition, existing survey data on unsafe behavior of construction workers mostly use cross-sectional surveys. To better identify unsafe behavior of construction workers, a field observation study was organized to improve the reliability of the results.

In summary, this study introduces Fuzzy-Set Qualitative Comparative Analysis (QCA) into the study of unsafe behavior of construction workers. Based on the theoretical framework of social cognition, this paper explores the influence of the combination of safety attitude, safety motivation, institutional control, safety climate, and safety training on the unsafe behavior of construction workers and verifies the existence and characteristics of the configuration effects of various factors affecting the unsafe behavior of construction workers, and explores the core and non-core conditions affecting construction workers, and tries to summarize multiple paths to improve safety performance.

## Theoretical Analysis and Research Framework

### Theoretical Analysis

#### The Unsafe Behavior of Construction Workers

The unsafe behavior of construction workers refers to the behavior that construction workers have violated the safety production system, safety operation methods, production technology regulations, and other behaviors that may lead to safety accidents in work engineering (Amponsah-Tawaih and Appiah, 2016). According to the definition of unsafe behavior of construction workers, it includes four meanings at two levels. Whether the violation of regulations is intentional or unintentional:①It can be divided into intentional violation of behavior regulations by workers and unintentional violation of behavior regulations by workers. At present, unsafe behaviors are largely caused by people violating safety regulations. Therefore, the point of this paper is to explore whether workers deliberately violate the rules of conduct (Martínez-Córcoles and Stephanou, 2017) and whether the cause of the accident is direct or indirect. ② Unsafe behavior that directly lead to accidents and unsafe behavior that indirectly lead to accidents. The performance that directly leads to unsafe behavior is that there is no safety defense against dangerous source, such as people sitting on the area with falling risk. Unsafe behaviors that indirectly lead to accidents include non-participation in safety education and training. Since unsafe behaviors that directly lead to accidents will immediately cause safety accidents, which are the main correction objects of safety management (Austin et al., 1996; Choudhry, 2008), this paper focuses on “unsafe behaviors that directly lead to accidents” as the main observation object.

In term of the identification of unsafe behaviors from the cognitive perspective, the existing research methods are mainly through the traditional questionnaire survey method. Considering the implicit nature of psychological cognition, some scholars have obtained data through interviews (Jiang et al., 2015) and behavioral observation methods (Kim et al., 2017) to explore the relationship between cognition and unsafe behavior. With the development of computer technology, some scholars applied computer science to safety management. For example, Choi et al. (Choi and Lee, 2018) built artificial intelligence to simulate the cognitive process of construction workers at the construction site, the relationship between environmental impact and safety behavior, and to explore the avoidance of unsafe behavior. Jokkaw et al. (2017) simulated high-rise building guardrails by virtual environment (VE) technology to study the relationship between cognition and unsafe behavior.

In order to explore the main reasons of construction workers’ unsafe behavior, scholars have carried out a large number of studies (Meng et al., 2021). The main psychological cognitive factor is safety attitude (Burns and Conchie, 2014; Shin et al., 2014; Man et al., 2019), safety motivation (Panuwatwanich et al., 2017; Xu et al., 2018), working pressure (Chang et al., 2005; Duma et al., 2014) and major institutional environmental factors including safety climate (Zhou et al., 2011; Liao et al., 2014; Guo et al., 2016b; Fargnoli and Lombardi, 2019), institutional regulation (Choudhry, 2014; Li et al., 2015) and education and training (Langford et al., 2000). These scholars hold different views on the key factors that influence the construction workers’ unsafe behavior. Specifically, when construction workers were more focused on construction sites and work with a positive and safety attitude, the incidence of unsafe behavior incidents was significantly reduced (Hasanzadeh et al., 2018). This conclusion is also supported by Zhang et al. (2020a), who found that improvement of attention, safety attitudes, and intrinsic motivation of construction worker may promote construction workers’ safe behavior, avoid and improve their risk perception. In addition, Fargnoli and Lombardi (2019) believe that promoting the behavior of construction workers should start with safety management, and formulating reasonable safety regulations that can create a good safety climate, thereby improving the construction workers’ unsafe behavior. Nicole and François (1991) also emphasized the importance of safety training and safety systems in creating a safety climate. Cavazza and Spape tested this point, arguing that the use of appropriate safety education and training can improve construction workers’ ability to identify risks and improve construction workers’ unsafe behavior. However, unlike Nicloe et al., and Cheng et al., found that unsafe behavior was often intentional (Cheng et al., 2022). Zhou et al. supported this view and found that safety training and safety systems are ineffective in creating a safety climate. What needs special concern is that good safety commitments are significantly related to safety climate (Zhou et al., 2011). Meanwhile, Zhang et al. (2020b) argued that safety culture should be a key factor in determining worker safety behavior. In summary, we can find that there is no unified view about the Causal conditions that affect the unsafe behavior of construction workers. In addition, psychological cognition and institutional environment play an important role in improving the unsafe behavior of construction workers. However, the existing studies are limited to a certain level of institutional environment or psychological cognition, and it is not clear how the synergistic effect of the two levels of elements affects the behavior of construction workers. This ignores that the construction workers’ unsafe behavior is a synergistic effect of multiple causal relationships and leads to inconsistent conclusions of existing studies (Petersen, 1971). In fact, unsafe behavior is a complex process that is influenced by the interaction between individuals and institutional situations. Therefore, in-depth research on the relevant important factors at the level of psychological cognition and institutional environment is an effective method to study the construction workers’ unsafe behavior.

#### Psychological Cognition Level

##### Safety Attitude and Unsafe Behavior of Construction Workers

Cheyne et al. (1998) defined safety attitude as the cognition of construction workers on the importance of safety in production, and the emotion of implementing safety policies, and the commitment to implementing safety rules and regulations. It is a reflective tendency to avoid safety accidents in production, or construction workers’ own beliefs and emotional tendencies about safety policies, management, and practices (Rundmo and Hale, 2003; Neal and Griffin, 2004). Safety attitude reflects construction workers’ positive or negative evaluation of the results of implementing safe construction behaviors and is a key factor in predicting behavioral intentions (Ajzen, 1991). On this basis, Shin et al. proposed the psychological process of safety behavior, and believed that behavior would give feedback on safety attitude, namely (risk perception→safety attitude→intention→behavior→risk perception). The construction workers obtained risk perception through their own knowledge. First, they established their own attitude, and then, workers judge whether to take some actions according to their own safety attitude, formed intentions, and obtained behavior results. Then, they feedback the risk perception according to behavior results and finally formed a safety attitude and form a feedback loop (Shin et al., 2014). Henning et al. believed that there was differential impact between occupational safety attitudes and different construction workers (Henning et al., 2009).

With the deepening of study on safety attitude, many antecedent mechanism models of different attitudes and behaviors have been developed. Among them, information processing theory and planned behavior theory have strong explanatory power on the relationship between safety attitude and behavior and believed that individual or environmental factors must affect behavior through attitude. Empirical studies also showed that the better the individual’s safety attitude, the greater the possibility of safety behavior (Ulleberg and Rundmo, 2003).

##### Safety Motivation and Unsafe Behavior of Construction Workers

Safety motivation refers to the willingness of construction workers to carry out construction in a safe way, showing the motivation of safe behavior (Griffin and Neal, 2000). In 1978, Andriessen first proposed the relationship between safety motivation and safety behavior, emphasizing that safety motivation affects safety behavior through reward and punishment (Andriessen, 1978). In 2000, Neal et al. verified that safety motivation can affect safety behavior through empirical research (Neal et al., 2000). Thus formed the view that the external environment as the core influence unsafe behavior. Regarding the issue of safety motivation affecting unsafe behavior, safety motivation is divided into two categories according to self-determination theory (Ryan and Deci, 2008): The first is “controlled safety motivation,” which considers that individuals due to the external environment motivation to do something under pressure, such as rewards and subsidies and institutional control; the second is “autonomous safety motivation,” which considers the motivation of individuals to do something out of their own choices, such as personal beliefs and hobbies. Recent studies have shown that autonomous safety motivation can promote individual’s positive pursuit of goal, while controlled safety motivation is not associated with the pursuit of individual goal (Koestner and Hope, 2014). At the same time, they have significant differences in the performance of safety behavior. It is generally believed that autonomous safety motivation can promote workers’ safety behavior (Scott et al., 2014; Jiang and Tetrick, 2016),while controlled safety motivation is considered to have no significant effect on safety behavior (Conchie, 2013), and even negatively affect safety behaviors (Jiang and Tetrick, 2016). Therefore, this study follows the mainstream viewpoint of selecting autonomous safety motivation for research.

Safety motivation is an important condition for construction workers’ safety behavior and has a predictive effect on construction workers’ safety behavior (Panuwatwanich et al., 2016). Because construction workers with higher safety motivation are more recognized for safe work and have higher self-efficacy at work, they are more likely to produce safety behaviors (Rigby and Ryan, 2018). At the same time, these safety behaviors can improve work recognition and self-efficacy, thereby promoting the generation of safety behaviors. Empirical studies have also shown that safety motivation can significantly improve safety behaviors (Kim et al., 2018).

#### Institutional Environment Level

##### Institutional Control and Unsafe Behavior of Construction Workers

Institutional control, proposed by North in (NIOSH (National Institute of Occupational Safety and Health), 2015), refers to corporate regulations, laws and regulations, and government policies that promote or restrict specific behaviors (Busenitz et al., 2000). Institutional control in the field of safety is mainly reflected in the fact that enterprises provide construction workers with excellent construction safety management systems and management processes to reduce the risk of safety accidents for construction workers, which is identified as an important factor in avoiding unsafe behavior of construction workers (Mohamed, 2002; Fam et al., 2017).

Good institutional control not only helps to reduce the unsafe behavior of construction workers, but also fully mobilizes the enthusiasm of construction workers (Jitwasinkul et al., 2016). In addition, substantial safety oversight may help improve the construction workers’ unsafe behavior (Fang et al., 2015). It is worth noting that immutable safety regulations are not enough, as Fam et al. (2017) propose that staff non-compliance with the regulations is due to unreasonable regulations, and therefore these regulations need to be timely reviewed and updated. This was verified by Iyer et al. (2004), who believed that project managers should communicate and interact more with construction workers, so as to better promote the order and safety of the construction site, and obtaining corporate support means construction workers gain legal status (such as sufficient working hours), and access to corresponding resources (including safety equipment and salary), not only create a better safety climate for the construction site, but also reduce the occurrence of safety accidents (Kim et al., 2019). The empirical study also shown that having an excellent safety management system indicates that management pay more attention to safety management, and provides institutional guarantee for construction enterprises to carry out work and safety training. This allows various stakeholders (such as investors and construction workers) to transmit positive signals of high safety performance of enterprises, which will make construction workers feel protected, and their safety behavior is naturally improved, so as to promote the interaction between institutional regulation and safety behavior.

##### Safety Training and Unsafe Behavior of Construction Workers

Safety training refers to the effective training for project personnel to achieve safe production, which usually including training to improve the safety production knowledge, skill level, and overall comprehensive quality of construction workers. The elements of safety training in the safety field reflect the popularity of safety knowledge in construction companies. It highlights whether managers are committed to effective training interventions (Weidman et al., 2015).

Lack of safety knowledge is an important reason for the unsafe behavior of construction workers (Choudhry and Fang, 2008). In the construction site, if construction workers have sufficient safety knowledge, they can fully identify potential risk factors and provide the ability to respond to risks in a timely manner. Can optimize safety management procedures. Construction workers mainly improve safety knowledge through accident occurrence and safety training. However, some existing safety trainings are mere formalities and cannot effectively deliver knowledge to construction workers. Limited by the frequency of safety training, it is difficult to guarantee and inefficient. Teaching methods and failure to fully motivate construction workers to learn, etc. (NIOSH (National Institute of Occupational Safety and Health), 2015). Effective safety training is therefore considered an important source of safety knowledge (Toole, 2002). If enterprises ensure that a certain frequency and effective to provide construction workers with a large number of safety accidents on how to cause and share the key points of specific safety construction, construction workers can not only learn more safety knowledge, industry knowledge to improve their professional quality and enhance their ability to deal with accidents, timely identify and eliminate potential risks, but also may stimulate their awareness of safety behavior as a career choice, so as to promote the smooth development of safety construction. On the contrary, if the safety training is difficult to guarantee the quality and quantity, the lack of reporting on the causes of safety accidents and explaining the conceptual knowledge of safety construction, construction workers usually lack the relevant knowledge and skills of safety construction, and even cannot identify risks and deal with safety accidents in time, nor will safety behavior as a professional idea, then safety construction will be hindered. More importantly, high-quality safety training system can provide an excellent platform for construction workers who are eager for safety, which helps construction workers better identify risks, enhance safety awareness, and thus generate safety behaviors. The research results have also been empirically verified that safety training can promote the construction workers’ safety behavior.

##### Safety Climate and Unsafe Behavior of Construction Workers

Safety climate originates from organizational climate. Organizational climate believes that various factors of working environment help to improve employees’ perception of working environment. According to organizational climate, Zohar (1980) defined safety climate as a common perception of workers’ safety working environment, focusing on the understanding of construction workers’ practices, procedures, and policies in the workplace. Some scholars believe that if individuals have a good understanding of safety, the probability of unsafe behavior on the construction site will decrease. Therefore, the safety climate can lead to changes in behavior and mentality, which is a key factor to improve safety performance (Fargnoli and Lombardi, 2019). However, with the deepening of the research on safety climate, the common measurement dimensions of safety climate have developed many dimensions and levels, including safety system, risk, work pressure, safety cognition, safety communication, management commitment, and so on (Guo et al., 2016b; Chen et al., 2017). Among them, the above research objectives are mostly organizations, supervisors, and colleagues, emphasizing the communication between the three. Shen et al. (2015) even believed that the safety climate is the behavior of guiding workers to adapt to the working environment. There is an interactive way of communication between the project teams to guide and ensure the safe construction.

If the construction workers on the construction site support and attach importance to safe construction, and regard safe construction as an ideal occupational requirement, at the same time, the role model effect of colleagues in the project and the leadership role in safety orientation will enhance individual safety behavior intentions, which will also stimulate potential construction workers. Workers’ sense of responsibility, enhance the self-efficacy of safe behavior, and better identify potential risks in the construction process, thereby improving the unsafe behavior of construction workers. In contrast, in a project with a low safety climate, people’s recognition of safety behavior and safety awareness is low in the whole project, and there is a lack of safety communication among colleagues and a cultural atmosphere to guide safe construction, and unsafe behaviors will easily breed. Empirical studies have also verified that the safety climate has a significant role in promoting the construction workers’ safety behavior. The better the safety climate, the easier it is to produce safe behaviors (Shea et al., 2021), and the safer communication among colleagues, the less likely to produce unsafe behaviors (Zamani, 2020).

### Study Framework

After reviewing the relevant literature, it is found that the current research on the unsafe behavior of construction workers has been continuously deepening and developing. In the research on the Causal conditions affecting the unsafe behavior of construction workers, scholars mainly discuss from the psychological cognitive level and the institutional environment level. The effects of safety attitude, safety motivation, institutional regulation, safety climate, and safety training on the unsafe behavior of construction workers are analyzed. In terms of research methods, regression analysis methods are mostly used, focusing on the “net effect” of a single factor on the unsafe behavior of construction workers, while ignoring the “chemical effect” that may exist among multiple factors.

The unsafe behavior of construction workers directly affects the safety performance of enterprises. With the frequent occurrence of construction safety accidents, social pressure and economic disputes prompt enterprises to carry out management reforms. Avoiding unsafe behavior of construction workers is an important challenge for enterprises. Especially in the face of the complexity and diversity of construction sites, the work flow and structural characteristics of different construction sites are different, among which construction workers have different safety attitudes and safety motivations, institutional control requirements and construction site safety climate are also different. Therefore, it is not clear how to coordinate the elements of psychological cognition and institutional environment to effectively avoid unsafe behavior of construction workers. When solving complex management problems, there will be many Causal conditions. The coupling of these Causal conditions forms different configurations to determine whether unsafe behavior occurs. This cannot be analyzed by traditional regression methods. The fsQCA based on holism can analyze the combination of multiple causality, which provides a new method to solve such problems with complex causality.

**Figure 2:**
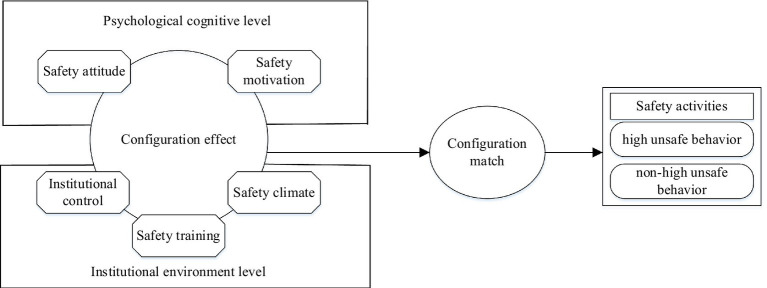
Conceptual model for configuration study of unsafe behavior influence mechanism of construction workers.

Bandura and Cervone (1986) focused on the role of human behavior in triggering behavior through observational learning and self-regulation, and proposed a more systematic theory of social cognition, pointed out that the generation of individual behavior was affected by environmental and cognitive factors. After it has been widely verified in the field of social psychology, many scholars have conducted research and applied it to other management fields, such as individual behavior, teaching reform, and organizational innovation to further verify and support this theory. Social cognitive theory mainly includes three aspects, namely, behavior, cognition, and environment. Among them, individual behavior, cognition, and environmental factors are not completely separated, but there is an internal interaction among the three (Higgins, 1995). Specifically, it means that an individual obtains information from the external environment and constructs self-cognition based on it. The individual’s intention and attitude determine the individual’s behavior, and the behavior will be consistent with the external environment, which in turn affects the individual’s intention and attitude. Therefore, how to carry out the linkage between psychological cognition and external environment needs to be further explored through configuration analysis. Based on the theoretical framework of social cognition and the above theoretical analysis, this study explores how the five Causal conditions of safety attitude, safety motivation, institutional control, safety training, and institutional environment are linked and matched from the perspectives of psychological cognition and institutional environment. How to avoid the occurrence of unsafe behavior as much as possible needs to be further analyzed through the configuration perspective and the fsQCA method. Therefore, a theoretical framework that affects the unsafe behavior of construction workers is constructed, as shown in Figure 2.

## Research Methods and Design

### Research Methods

Qualitative Comparative Analysis (QCA) research method was first proposed by American sociologist Charles Ragin. Due to its advantages of case comparative analysis and quantitative research, the method has been widely recognized and widely used in sociology, political science and management, and other disciplines. (Llopis-Albert et al., 2019; Park and Mithas, 2020; Du and Kim, 2021). As an important tool for solving complex relationships (Fiss, 2007), the QCA method is especially suitable for small and medium sample data. Based on set theory and Boolean algebra, it explores how the combination of Causal conditions leads to changes in complex results. At present, there are four kinds of QCA methods: Crisp-set QCA (csQCA), Fuzzy-set QCA (fsQCA), Multivalue QCA (mvQCA), and tTemporal QCA (tQCA). Among them, the csQCA method mainly deals with dichotomous variables, while the mvQCA method allows multi-valued variables, and the fsQCA method combines the principles of fuzzy logic, which can not only deal with dichotomous and multi-valued variables, but also continuous data. It is an extended version of csQCA and mvQCA. The tsQCA method mainly studies dynamic variables. Considering that the Causal conditions and outcome variables of this research involve degree and category issues, that is, there are both clear sets and fuzzy sets, the widely used fsQCA method is finally selected for analysis.

This study employs the fsQCA approach to explore the causal complex mechanisms driving unsafe behavior of construction workers, mainly based on the following reasons:①The unsafe behavior of construction workers is the result of the combined action of various elements of psychological cognition and institutional environment. Using this method, the non-linear relationship between each element and unsafe behavior can be explored. ②The research problem of this paper is to explore multiple equivalent paths of unsafe behaviors based on those factors that can affect the occurrence of unsafe behaviors. ③This paper focuses on the antecedents of unsafe behaviors of construction workers. This method can compare the asymmetric antecedents of construction workers’ high unsafe behaviors and non-high unsafe behaviors, and deepen the research conclusions.

There are also two important parameters in QCA methodology, consistency and coverage, which are explained as follows:

Consistency: In order to test the fit degree of antecedent condition combination and another set, that is, the consistency degree of the influence of condition variable combination on the result variable, the calculation formula is as follows:


$$\mathrm{Consistency}\left(X_{i}\leq Y_{i}\right)=\sum \min\left(X_{i}{\mathrm{,Y}}_{i}\right)/\sum X_{i}$$

Coverage: it is used to evaluate the coverage degree of the combination of antecedent conditions on the result variables. The calculation formula is as follows:


$$\mathrm{Coverage}\left(X_{i}\leq Y_{i}\right)=\sum \min\left(X_{i}{\mathrm{,Y}}_{i}\right)/\sum Y_{i}$$

where X_i_ represents the membership degree in the combination of conditions; Y_i_ represents the membership degree in the result variable; Both values are in the range of (0,1).

**Figure 3:**
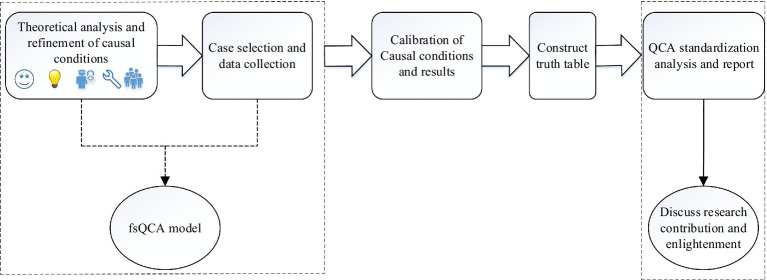
The main steps of fsQCA method.

Generally speaking, fsQCA mainly includes the following five steps (as shown in Figure 3): ①Theoretical analysis and refinement of Causal conditions; ②Case selection and data collection; ③Calibration of Causal conditions and results; ④Construct truth table; ⑤QCA standardization analysis and report; ⑥Discuss research contribution and enlightenment.

### Research Design

#### Questionnaire Collection and Recovery

##### Unsafe Behavior Data Collection

The theme of this study is unsafe behavior of construction workers, and the sample selection is mainly aimed at front-line construction sites and large-scale construction enterprises. Data collection will be carried out from September 2021 to November 2021, and the data are from four cities in China, including Ganzhou, Luoyang, Changsha, and Zhengzhou. The selected construction sites are all in the construction stage and the number of people in the project is not less than 70. A total of nine construction companies are investigated, and the research data is diversified.

According to the results of the accident investigation and report of the Ministry of Housing and Urban–Rural Development of the People’s Republic of China (Safety Production Management Committee), the types of construction safety accidents are mainly composed of high-altitude fall (53.69%), object strike (15.91%), earth, foundation pit collapse (8.93%), and hoisting machinery damage (5.43%). Based on the above four main potential risks, the observation scale of unsafe behavior of construction workers was developed by reading and combing relevant academic literature (Mohajeri et al., 2021), safety management manual, and expert interviews of a well-known enterprise in China. On this basis, the site observation was carried out at the construction site to understand the work content and types of work in each region of the site. Then, the project managers at each site are exchanged and discussed, and the unsafe behavior scale of construction workers is revised to obtain the final list. The list mainly includes14 unsafe acts in four broad categories, such as climbing and sitting in areas with a risk of falling, entering the site without wearing protective equipment, such as helmets, and prematurely removing formwork or supports, as shown in Table 1. At present, most existing studies use self-reported questionnaires to measure unsafe behavior of construction workers, which may lead to large measurement deviations (Li et al., 2017). Therefore, this study measures workers’ unsafe and safe behaviors according to the observation scale “on-site observation,” and divides the safety behaviors of construction workers into dichotomous variables “unsafe behaviors” and “safe behaviors,” as shown below.


$$Y=\left\{\begin{array}{c} 1,\mathrm{Workers observed to exhibit unsafe behavior}\\ 0,\mathrm{others}\;\;\;\;\;\;\;\;\;\;\;\;\;\;\;\;\;\;\;\;\;\;\;\;\;\;\;\;\;\;\;\;\;\;\;\;\;\;\;\;\;\;\;\;\;\;\; \end{array}\right.$$

**Table 1:** Safety observation scale for Construction Worker behavior.

No.	Type of accident risk	Specific unsafe behaviors
1	High falling	Sitting in areas at risk of falling (such as railings and scaffolding).
		Using incorrect climbing tools (such as material lifting device).
		In the process of erecting scaffolding and steel support, the platform is unsafe and no safety belt is used.
		Unauthorized removal of safety protection devices.
2	Object strike	Personal protective equipment, such as safety hats not worn into site.
		Transmission of tools and materials at high places.
		No safe passage on construction site.
3	Earth, foundation pit collapse	Safety measures, such as premature removal of formwork or support.
		Entering the pit from the edge of the pit with large slopes or obstacles.
		Do not set up scaffolding as required.
4	Lifting machinery damage	Wearing gloves to command or operate slings, or multiple people to command, without standard gestures.
		The lifting operation through the personnel area or the operation area does not ring the flute.
		Maintenance, cleaning, maintenance, and so on during mechanical operation.

Note: Y = 1 means the worker’s behavior is unsafe, Y = 0 means the worker’s behavior is safe.

The specific operations are as follows: First, 15 observers with rich construction experience are selected, and samples of workers with unsafe behaviors need to be selected on the spot according to the unsafe behavior observation scale. Before starting the investigation, the observer informed each construction worker that the purpose of the study is from academic research. There are no business dealings with construction companies. The survey results will help construction workers in a safer working environment, reassure workers, and gain support from construction workers. Secondly, considering that the presence of observers may lead to deviations in workers’ behavior, observers are required to try their best not to observe and record at the first site. It is found that people’s potential behavioral deviations toward the presence of observers are reduced, their behaviors tend to be routine, and the treatment methods are approved by the person in charge of the scene. Finally, the recorded observations are matched with the questionnaire results of the same construction workers.

#### Questionnaire Development and Measurement

On the basis of existing research, the social cognition theory is used as the research framework, combined with the context of safety practice in China’s construction industry. This paper mainly determines the questionnaire with 17 items from the following five dimensions: safety attitude (3 items), safety motivation (4 items), institutional control (3 items), safety training (3 items), safety climate (4 items). The questionnaire is adapted from the study by Brondino et al. (2012); Fang et al. (2015); Jiang et al. (2015); Jiang and Tetrick (2016), and Alruqi et al. (2018). Before the formal survey, a preliminary survey of construction workers on the construction site is conducted. And based on the feedback from the pre-investigation, the questionnaire is adjusted accordingly to ensure that the questionnaire questions are clearly stated.

Questions are conducted through structured interviews with construction workers, and forms are filled out based on worker responses. All items are based on Likert 5 level scale (1 = strongly disagree, 2 = disagree, 3 = average, 4 = agree, 5 = strongly agree). Table 2 lists the study variables, including their descriptions, means, and standard deviations.

**Table 2:** Variable measurement and descriptive statistics.

Variable	Variable description	Average value	Standard deviation
Safety motivation	Adopting safe behaviors helps me accomplish my work tasks.	4.35	0.95
	Adopting safe behaviors in order to be able to become your ideal self at work as quickly as possible.	4.30	1.07
	Adopting safe action is because I enjoy the whole process of effort.	4.24	1.11
	Adopting safe behavior is because it can bring me satisfaction.	4.27	1.09
Safety attitude	Accidents at work are inevitable.	3.52	1.52
	I can also do the work of security personnel, which is relatively simple.	0.27	1.43
	If the safe operation rules are convenient and feasible, it can promote my safe work.	3.63	1.33
Institutional control	The company will regularly organize security assessments.	3.84	1.14
	If I don ‘t have protective equipment (like helmets), my supervisor will scold me.	3.91	1.14
	Safety guards supervise staff behavior at construction site.	3.97	1.15
Safety training	My company trains employees on workplace safety issues.	4.11	1.18
	Give me safety training enough to assess workplace hazards.	4.10	1.00
	Management encourages us to attend security training courses.	4.09	1.20
Safety climate	Management takes corrective action against unsafe measures.	4.14	0.98
	Team members provide guidance for security work.	3.95	0.90
	Team members remind the use of safety equipment.	4.00	0.89
	Team members discuss security risks.	3.71	1.04

*The values of potential variables in Table 2* *are equal to the mean values of all observed variables*.

#### Descriptive Statistical Analysis

A total of 180 questionnaires were sent out in the study, with an average of 20 at each site. A total of 164 valid questionnaires were collected, with an effective recovery rate of 91.1%, excluding those with incomplete interviews and chaotic logical expression. The sampling process can be considered as simple random sampling because construction workers are selected by random observation in the project. In addition, prior to the interview, in order to improve the validity of the case, ① the workers were informed in advance that the study was only academic; ② Interview results will be anonymized; ③ Interview questions should be concise and easy to understand.

The descriptive statistics of the survey respondents are shown in Table 3. The local construction workers are mainly male, which is in line with the actual situation of the construction site. The sample age distribution is: less than 25 years old, 12.8%, 25–35 years old, 23.8%, 35–45 years old, 32.9%, and over 45 years old, 30.5%. The proportion of construction workers over the age of 35 accounts for 63.4%, which is in line with the “aging” population characteristics of today’s construction sites. The education level of most workers is lower than the high school level (68.9%), which also shows the reality of construction workers’ low level of education. Among them, most of the workers have more than 10 years of work experience, accounting for 60.4% of the workers. It can be seen that the survey respondents have relatively rich work experience. In addition, the distribution of various types of work is relatively uniform. To sum up, the work experience, age, and education level of the respondents in this study are in line with the distribution of the construction industry population, so the selection of the survey sample is representative.

**Table 3:** Demographic characteristics of construction workers (*n* = 164).

Characteristics	Items	Frequency	Percentage (%)
Gender	Male	130	79.3
	Female	34	20.7
Age	<25	21	12.8
	25–35	39	23.8
	35–45	54	32.9
	>45	50	30.5
Work experience	<5	18	10.9
	5–10	47	28.7
	10–15	49	29.9
	>15	50	30.5
Education	Primary school or below	45	27.4
	Secondary school	68	41.5
	Senior high school	34	20.7
	Bachelor’s degree or equivalent	17	10.4
Type of work	Steel Fixer	44	26.8
	Solid Plasterer	27	16.5
	Scaffolder	34	20.7
	Special type operator	44	26.8
	Others	15	9.1

### Common Method Bias

Considering that the obtained data are mostly perceptual, the collected sample data may have a common method deviation. In order to avoid common method deviation, this study uses some reverse items and different time measurement facilities to control common method deviation from the program. At the same time, this paper draws on the method of Liang et al. (2007), and adopts the Harman single factor method to test the common method bias. In the factor analysis without rotation, five principal components with eigenvalues greater than 1 are extracted, and the maximum principal component explained 35.11% of the total variance, which is lower than 40%, indicating that the common method deviation in this study is within an acceptable range.

### Reliability and Validity Analysis

This study uses SPSS. 24.0 to test the reliability of the sample data. Firstly, the overall reliability of the questionnaire is analyzed. The overall Cronbach’s alpha value is 0.921, indicating that the overall reliability of the questionnaire is good. Then analyze the reliability of different variables. It is found that the minimum value of Cronbach’s alpha coefficient of each variable is 0.801. The threshold of Cronbach’s alpha greater than 0.7 is met. It shows that the survey measurement item has high reliability and can reflect each variable accurately (Hair et al., 2011), as shown in Table 4.

**Table 4:** Reliability and validity analysis results.

Potential variable	Observational variables	Factor load value	Cronbach’s alpha	CR	AVE
Institutional environment	IE1	0.889	0.827	0.825	0.615
	IE2	0.790			
	IE3	0.657			
Safety attitude	SA1	0.772	0.859	0.864	0.681
	SA2	0.942			
	SA3	0.748			
Safety training	ST1	0.860	0.882	0.888	0.725
	ST2	0.781			
	ST3	0.909			
Safety motivation	SM1	0.838	0.921	0.924	0.754
	SM2	0.912			
	SM3	0.920			
	SM4	0.796			
Safety climate	SC1	0.579	0.801	0.822	0.549
	SC2	0.874			
	SC3	0.895			
	SC4	0.543			

This study further tests the validity. Firstly, KMO test and Bartlett spherical test are used to test the questionnaire. The KMO value is 0.867, which is higher than 0.8, and the explicitness (0.000) is less than 0.05, so it is suitable for factor analysis. In terms of validity testing, the most commonly used Convergent Validity and Discriminant Validity tests are used to measure the comprehensiveness and exclusivity of variables, respectively, (Guo et al., 2019). Referring to the suggestion of Fornell and Larcker (1981) and Hair et al. (2011), the average variance extraction (AVE), combined reliability (CR), and factor loading values of latent variables are used to test the convergence validity. The latent variable factor loading value corresponding to the measured variable is required to be greater than 0.5, and the CR value is greater than 0.7. The factor loading values in the study are all between 0.543 and 0.942, and the CR values of each variable are all greater than 0.7, indicating that each dimension has a high internal consistency. According to the proposal of Hair et al. (2011), the acceptable range of AVE value is 0.36–0.50, and greater than 0.5 is ideal. The AVE value of each variable in the study is greater than 0.5, indicating that the research dimension can explain the variance of the variable well and has good convergent validity.

As shown in Table 5, the discriminant validity test needs to satisfy that the AVE arithmetic square root of all latent variables is greater than the correlation coefficient between the variable and other latent variables (Chen et al., 2016). The diagonal line of Table 5 is the square root of AVE corresponding to each latent variable. It is found that the correlation coefficients of the five research variables meet the requirements. At the same time, the QCA method emphasizes configuration thinking, which is different from the traditional method that believes that variables must be independent of each other, and recognizes the existence of correlation between variables (Pappas et al., 2016). Therefore, the validity test standard can be appropriately reduced, indicating that the collected data has good discriminant validity.

**Table 5:** Correlation and differential validity of the variables.

	Institutional environment	Safety attitude	Safety training	Safety motivation	Safety climate
Institutional environment	**0.784**				
Safety attitude	0.503	**0.825**			
Safety training	0.436	0.234	**0.852**		
Safety motivation	0.343	0.264	0.820	**0.868**	
Safety climate	0.335	0.209	0.740	0.706	**0.741**

The significance test of the correlation coefficient all meet ^**^p < 0.01.

## Empirical Analysis

### Variable Calibration

The fsQCA method first requires calibration of the variables to form a fuzzy set ranging from 0 to 1 (Ragin, 2008). The data types of condition variables and outcome variables in the questionnaire are divided into two categories: (1) Dichotomous data: unsafe behavior of construction workers; (2) Scale data: safety climate, safety motivation, institutional environment, safety attitude, safety training, and other five Causal conditions. For the calibration of binary data, the observed unsafe behavior is assigned a value of “1,” the safe behavior is assigned a value of “0,” and the data is a binary variable of 0 or 1. It conforms to the membership scale between 0 and 1 and satisfies the fsQCA Boolean logic analysis conditions. The data of the scale needs to be calibrated and transformed. Due to the lack of corresponding external and theoretical calibration for the measurement of Causal conditions, such as the institutional environment and safety attitude in this paper, a direct calibration method is adopted with reference to previous studies (Greckhamer, 2016). At the same time, considering the non-normal distribution of 5 antecedent condition data, referring to the calibration method of Wu et al. (2019), the three calibration points of complete membership, intersection, and complete non-membership were set as the quartile (75%), mean and lower quartile (25%) in the case sample description statistics. As shown in Table 6.

**Table 6:** Anchors of the set and calibration.

	Causal condition	Target set	Anchors		
			Full membership point	Cross over point	Full non-membership point
Conditional variable	Safety attitude (SA)	Excellent safety attitude	4.58	3.41	2.42
	Safety motivation (SM)	Excellent safety motivation	5.00	4.29	4.00
	Institutional environment (IE)	Excellent institutional control	4.67	3.91	3.42
	Safety training (ST)	Excellent safety training	5.00	4.10	3.67
	Safety climate (SC)	Excellent safety climate	4.50	3.95	3.50

Then, the data is calibrated by fsQCA3.0, and the questionnaire data is converted into a fuzzy membership degree between 0 and 1. Some Causal conditions are calibrated to generate an intersection of 0.5. In order to avoid errors, it can be calculated and classified. Using Fiss’s suggestion (Fiss, 2011) to add a constant 0.001 to the intersection, the calibration results of the Causal conditions and the outcome variables are finally obtained, as shown in Table 7.

**Table 7:** Variable calibration results (part).

CASE	SA	SM	IE	ST	SC	UB
1	0	0.01	0.95	0.95	0.99	1
2	0	0.01	0.95	0.95	0.99	1
3	0	0.22	0.95	0.95	0.99	0
4	0	0.22	0.95	0.95	0.99	0
5	0.03	0.82	0.05	0.95	0.01	1
6	0.19	0.91	0.95	0.87	0.57	1
7	0.45	0.501	0.5	0.501	0	0
8	0.59	0.82	0.95	0.95	0.99	0
9	0.59	0.82	0.95	0.95	0.99	0
10	0.84	0.98	0.95	0.71	0.99	0
11	0.84	0.44	0.95	0.95	0.95	0
12	0.95	0.91	0.68	0.87	0.95	1
13	0.95	0	0.87	0.95	0.84	1
14	0.95	0.1	0.87	0.95	0.84	1
15	0.95	0.96	0.68	0.87	0.84	0
16	0.95	0	0.87	0.87	0.84	1
17	0.99	0.98	0.95	0.95	0.95	0
18	0.99	0.04	0.68	0.4	0.95	1
19	0.99	0.98	0.95	0.4	0.95	0
20	0.99	0.98	0.87	0.4	0.01	0

### Necessary Condition Analysis

Before configuration analysis, it is necessary to check whether a single Causal condition constitutes a necessary condition for the outcome variable. When the consistency of the Causal condition reaches 0.9 (Schneider and Wagemann, 2012), the Causal condition is a necessary condition for the outcome variable. From the research data, as shown in Table 8, it is found that the unsafe behavior of construction workers with high and ~ high heights has no conditions to meet this standard, indicating that any single condition cannot constitute a necessary condition for the outcome variable. Therefore, the effect of the combination of Causal conditions on the outcome variable will be further analyzed.

**Table 8:** Necessity analysis of single Causal condition.

Causal condition		Outcome variable	
		High UB	Non-high UB
Psychological cognitive level	Safety attitude(SA)	0.34	0.85
	~Safety attitude(~SA)	0.66	0.15
	Safety motivation(SM)	0.53	0.77
	~Safety motivation(~SM)	0.47	0.23
Institutional environment level	Institutional environment(IE)	0.48	0.38
	~Institutional environment(~IE)	0.52	0.73
	Safety training(ST)	0.44	0.82
	~Safety training(~ST)	0.56	0.18
	Safety climate(SC)	0.44	0.73
	~Safety climate(~SC)	0.56	0.27

*“~” expresses “not” in logical expression*

### Build the Truth Table

Based on the fuzzy set membership score matrix, use fsQCA3.0 to calculate the truth table, and get all the theoretically appearing conditional configurations (2^5^). However, there may be some configurations in practice and there is no real case evidence. Therefore, a simplified operation is performed on the truth table composed of all conditional configurations. The simplification of the truth table usually requires setting thresholds on the case data, including the number of cases, consistency, and PRI consistency (Proportional reduction in inconsistency). Following the Fiss opinion, the case frequency threshold is set to 3 for samples with a number greater than 150 (Ragin, 2008; Fiss, 2011). Also referring to Rihoux and Ragin’s treatment, set the consistency threshold to 0.75 (Rihoux and Ragin, 2009). PRI consistency is used to avoid conflicting configurations in analysis results. Following the recommendation of Greckgamer et al., the threshold for PRI is set to 0.7 (Greckhamer et al., 2018). Finally, the configuration that meets the conditions is the fuzzy subset of the result, and the code is assigned a value of 1, and the configuration that does not meet the conditions is assigned a value of 0. The results are shown in Table 9.

**Table 9:** True table (part).

Causal condition					Number of cases	Outcome variable	Consistency	PRI consist
SA	SM	IE	ST	SC		UB		
0	0	0	0	0	19	1	0.92	0.92
0	0	1	0	0	5	1	0.85	0.85
0	1	1	1	1	20	1	0.82	0.82
0	1	1	1	0	4	1	0.81	0.81
0	0	1	0	1	3	1	0.79	0.79
0	0	1	1	1	5	1	0.77	0.77
0	1	0	1	0	3	1	0.77	0.77
1	1	0	0	0	3	1	0.77	0.77
1 0	0 1	0 0	0 1	0 1	6 13	0 0	0.75 0.72	0.75 0.72

### Sufficiency Analysis of Conditional Configuration

Configuration analysis can reveal the sufficiency of the results caused by different configurations of multiple antecedents. Through fsQCA fuzzy set analysis, complex solutions, parsimonious solution, and intermediate solution are obtained. Using Rihoux and Ragin’s suggestion (Rihoux and Ragin, 2008), the intermediate solution with moderate complexity close to the case is used as the reported configuration analysis result. At the same time, in order to effectively clarify the core conditions and marginal conditions of the combination of various Causal conditions for the unsafe behavior of construction workers, the parsimonious solution and the intermediate solution are compared (Fiss, 2011). Among them, the core condition is a factor of practical significance that appears in the intermediate solution and the simple solution, while the marginal condition only appears in the intermediate solution alone, and there is no example to prove it. Referring to the Fiss reporting paradigm, the configuration configurations of each condition are shown in Table 10 (Fiss, 2011). Overall, the consistency of the single and overall solutions of the six configurations presented in the table is higher than the 0.75 threshold suggested by Raigin. Taking high unsafe behavior as an example, its total consistency is 0.893, indicating that in all cases satisfying these 5 configurations, about 89.3% of construction workers exhibited unsafe behavior, indicating the validity of the results. At the same time, the total coverage of the solutions reached 0.5 and 0.476, indicating that 5 and 1 configurations explained about 50 and 47.6% of the cases, respectively. It shows that these six condition configurations have good explanatory power for the construction workers’ safety behavior. Since the presence and absence of each Causal condition may lead to unsafe behavior of construction workers, this study does not conduct counterfactual analysis and selects “presence or absence” for all conditions in the standardized analysis.

**Table 10:** Configuration of high and non-high construction workers’ safety behavior.

Causal condition	High unsafe behavior configuration					Non-high UB configuration
	S1	S2	S3	S4	S5	NS1
Safety attitude	⊗	⊗	⊗	⊗	●	●
Safety motivation	⊗	●	⊗		●	●
Institutional environment		●	●	●	⊗	●
Safety training	⊗	●		●	⊗	●
Safety climate	⊗	⊗	●	●	⊗	●
Consistency	0.926	0.814	0.829	0.820	0.766	0.774
Raw coverage	0.251	0.105	0.098	0.174	0.086	0.476
Unique coverage	0.183	0.034	0.014	0.083	0.051	0.476
Solution consistency			0.893			0.774
Solution coverage			0.500			0.476

*(1) ● represents the core condition exists, ● represents the edge condition exists. (2) ⊗ represents the lack of core condition, ⊗ represents the lack of edge condition. (3) space representation conditions may exist or not exist*.

The conditional variable combinations of five high unsafe behavior configurations are analyzed:

Configuration 1 (~SA × ~SM × ~ST × ~SC), the consistency of this configuration reaches 0.926, and the unique coverage is the highest among all configurations, reaching 0.183. Among them, the absence of safety attitude and safety climate plays a central role, the absence of safety motivation, and safety training plays an auxiliary role, and institutional control is an irrelevant condition. Configuration 2 (~SA × SM × IE × ST × ~SC), the consistency of this configuration reaches 0.814, and the unique coverage reaches 0.034. Among them, the lack of safety attitude and safety climate, the existence of safety motivation and safety training play a central role together, and institutional control plays an auxiliary role. Configuration 3 (~SA × ~SM × IE × SC), the consistency of this configuration reaches 0.829, and the unique coverage reaches 0.014. The absence of safety attitude and the existence of institutional control play a central role together, the absence of safety motivation and the existence of safety climate play an auxiliary role, and safety training is an irrelevant condition. For configuration 4 (~SA × IE × ST × SC), the consistency of this configuration reaches 0.820, and the unique coverage reaches 0.083. Among them, the absence of safety attitude and the existence of institutional control play a central role, safety training, and safety climate play an auxiliary role, and safety motivation is an irrelevant condition. Configuration 5 (SA × SM × ~IE × ~ST × ~SC) has a consistency of 0.766 and a unique coverage of 0.051. Among them, institutional control, the absence of safety climate and the existence of safety motivation play a central role, and safety attitude and safety training play an auxiliary role.

The combination of conditional variables of kind of non-high unsafe behavior configuration is analyzed:

Configuration 1 (SA × SM × IE × ST × SC), the consistency of this configuration reaches 0.774, and the unique coverage reaches 0.476. Safety attitude, safety motivation, safety training, and safety climate play a central role, while institutional regulation plays a supporting role, and the complementary combination of the five factors drives the avoidance of unsafe behavior of construction workers.

Overall, the generation of high and non-high unsafe behaviors of construction workers has causal asymmetry, and there are five different paths for the configuration of high unsafe behaviors. These five configuration paths are sufficient to explain the high unsafe behavior of construction workers. Condition, the combination of non-high safety attitude, non-high safety motivation, non-high safety training, and non-high safety climate conditions will be more likely to produce unsafe behavior of construction workers. For the non-tall unsafe behavior configuration, there is a path to account for the non-tall construction worker unsafe behavior. High safety attitude, high safety motivation, high safety training, and high safety climate avoid unsafe behavior of construction workers.

### Stability Test

This paper conducts a stability test on the antecedent configuration of unsafe behavior of tall construction workers (Judge et al., 2020) First, increasing the consistency threshold from 0.75 to 0.77, the resulting configurations are basically consistent. Second, increasing the PRI consistency threshold from 0.7 to 0.75 produces basically consistent configurations. Finally, considering that the samples from different cities and construction projects may have differences in resource endowments, after randomly deleting 15 cases from a certain project, the configurations obtained by the analysis are still basically the same, indicating that the research results meet the stability test standards (Thomas et al., 2018).

### Results and Discussion

The fsQCA effectively identifies six configuration paths that lead to high and non-high results, indicating that the unsafe behavior of construction workers is characterized by multiple factors concurrent and causal asymmetry. According to the core conditions contained in the six configurations and the theoretical logic behind the configurations. Combined with the actual situation, the internal mechanism of each configuration leading to the formation mode of unsafe behavior of tall and non-tall construction workers is summarized and analyzed, as shown in Table 11.

**Table 11:** Formation mode and theoretical summary of high and non-high result configurations.

Configuration path	Formation model	Theoretical support
~SA × ~SM × ~ST × ~SC	Attitude-climate scarcity type	The theory of planned behavior、Persuasion theory
~SA × SM × IE × ST × ~SC	Attitude-climate scarcity type	The theory of planned behavior、Persuasion theory
~SA × ~SM × IE × SC	Psychological cognitive scarcity type	Self-determination theory
~SA × IE × ST × SC	Psychological cognitive scarcity type	Self-determination theory
SA × SM × ~IE × ~ST × ~SC	Institutional environment scarcity type	Trait activation theory
SA × SM × IE × ST × SC	Comprehensive type	Individual–environment matching theory

#### High Unsafe Behavior Pattern of Construction Workers

##### Attitude-Climate Scarcity Type

Attitude-climate scarcity type is the intersection of configuration 1 and configuration 2. This type shows that when the safety attitude and safety climate of construction workers are at a low level, even if construction workers have a high level of safety motivation and good institutional control and safety training on projects, they will still lead to high unsafe behaviors of construction workers. Therefore, the construction workers with such characteristics are called “attitude-climate scarcity type.” In view of this type, the construction workers in the project generally show insufficient awareness of the importance of safety, and there is a deviation in the understanding of safety throughout the project. According to the theory of planned behavior proposed by Ajzen (1991), the influence of construction workers’ safety attitude on unsafe behavior is verified. TPB believes that individual behavior is affected by norms, attitudes, and intuitive behavior control. Attitude means that if the evaluation result of an individual after engaging in a certain behavior is negative, it will have a negative impact on this behavior. On the contrary, the more active the individual’s attitude toward a certain behavior, the stronger the willingness to implement the action. At the same time, the safety climate plays an important role in unsafe behaviors, and the persuasion theory is verified. It is believed that the communication in the group will indirectly affect the generation of individual behaviors. Especially in uncertain situations, individuals tend to obtain information from the outside world, and individual behaviors obey group norms (Eagly and Chaiken, 1993). Generally speaking, construction workers tend to be prone to unsafe behaviors when their attitude toward safety is at a low level. When construction workers have unsafe behaviors, considering that the group has a low common cognition of the importance of safety, they often do not correct the unsafe behaviors of individuals, but instead spread unsafe behaviors, resulting in an increase in unsafe behaviors of the project team. These groups tend to be younger employees with shorter tenures. In the actual interview, it is found that they have insufficient awareness of the importance of safety, and they are even accustomed to unsafe behaviors. At the same time, they also say that they have less contact with other employees and rarely communicates about construction safety. By comparing the other types, according to the coverage index, S1 and S2 jointly explained 35% of the results of high unsafe behaviors, and unsafe behaviors are more likely to occur, that is, most groups cause high unsafe behaviors through this type of behavior. This also fully shows that unsafe behavior is jointly influenced by cognition and system, and its influence on unsafe behavior even exceeds some formal institutional factors.

##### Psychological Cognition Scarcity Type

Psychological cognition scarcity type is the intersection of configuration 3 and configuration 4. This type indicates that when the safety attitude and safety motivation of construction workers are at a low level, regardless of the effect of the project on safety training for construction workers, even if the institutional control and safety climate of the project are at a high level, high unsafe behaviors will still occur. Therefore, construction workers with such characteristics are called “psychological cognitive scarcity type.” In this category, the safety attitude and safety motivation of construction workers are the core influencing factors. According to Self-determination theory, the judgment of construction workers whether to produce safe behaviors is a game between “effort” and their own “safety identity.” The more construction workers agree with the value and importance of safe work, the more they think the effort is worth it and can continue to spontaneously demonstrate safe behavior. At the same time, because people are independent individuals, their sustainable safe behavior can only be chosen by their own will. However, the safety behaviors produced by construction workers under environmental supervision are subject to greater external regulation. If there is no timely internal transformation (learning, etc.) into their own safety awareness, unsafe behaviors will often occur. From the specific situation, it is often the workers with higher working years and lower educational level who are in this configuration. Their willingness to actively learn is weak, and they show burnout at work, lack of enthusiasm for work, and lack of sense of achievement in work.

##### Institutional Environment Scarcity Type

Configuration 5 shows that when the institutional environment and safety climate of the project are at a low level, and there is a lack of good safety training. Even if the construction workers themselves have a high level of safety motivation and safety attitude, construction workers will have high unsafe behaviors. This group is the “institutional environment scarcity type,” in which the institutional control and safety climate of construction workers are the core influencing factors. According to Trait Activation Theory, situational elements, such as groups, organizations, and tasks affect the influence of traits on behavior, while traits are internal attributes of individuals and stable characteristics that describe individual behavior (Tett and Guterman, 2000). The process by which a trait affects behavior is a process in which a trait hidden within an individual is activated in an “appropriate” situation and manifests a specific behavior. As an important platform for safety behavior, the institutional environment directly affects the improvement and development of construction workers’ safety capabilities. A better safety climate and institutional control can make employees feel the management’s support for safety and enhance the willingness of construction workers to behave safely. And make managers and construction workers communicate and cooperate, enhance the trust between construction workers and management and the emotional connection between construction workers. Therefore, the institutional environment scarcity type is a path of construction workers’ highly unsafe behavior. Even if individuals have a high level of safety attitude and motivation, when the institutional environment matches with a lower level of institutional control, safety climate, and safety climate, it will still lead to unsafe behaviors of construction workers.

#### Construction Worker Non-highly Unsafe Behavior Patterns Comprehensive

Configuration 6 shows that when the individual’s safety attitude and safety motivation are at a high level and then match the project’s high level of institutional control, safety climate, after good safety training, construction workers will appear non-high unsafe behavior, such groups are called “comprehensive.” The configuration verifies that high insecurity can still occur if the administrator starts only from a single aspect (individual, environment) and should consider the multidimensional interaction. According to the original coverage rate of 0.476, it is found that 47.6 percent of the people surveyed belong to the comprehensive type, so the management should turn to the comprehensive type to the type of development struggle. According to the individual–environment matching theory, it is believed that people and the environment are both direct influencing factors of behavior, and the interaction between the two will have a strong impact on behavior (Edwards, 2008). The psychological cognition level and institutional environment level of workers are the important basis for the unsafe behavior of construction workers. At the same time, the theory of human–environment matching emphasizes the dynamic role and considers the dynamic interaction between the individual and the environment. That is, construction workers’ psychological cognition and institutional environment influence each other, and both aspects need to maintain a high level, which is more likely to produce non-high unsafe behaviors. On the whole, the formation of unsafe behaviors of construction workers is a complex process, which is not only affected by psychological cognition, but also affected by multiple factors, such as the institutional environment. Comparing the configurations that affect the high unsafe behaviors, it is found that the reasons affecting the unsafe behaviors of construction workers are asymmetric, that is, the non-high unsafe behaviors of construction workers are not the opposite of the high unsafe behaviors of construction workers. At the same time, comparing various Causal conditions, it is found that safety attitude and safety climate are missing as core conditions, which play a key role in the production of unsafe behaviors.

## Research Conclusions and Implications

### Research Conclusions

How to design effective management measures to intervene in the unsafe behavior of construction workers is the focus of construction safety research. Through field observation, this paper interviews and surveys 164 construction workers from 9 construction enterprises. Based on the SCT framework, starting from the psychological cognitive level and the institutional environment level, the fsQCA method, and configuration thinking are used to integrate the five elements of the above two levels, and to explore the causal and complex mechanisms that affect the unsafe behavior of construction workers. This study draws the following conclusions:

(1) It is found that any single antecedent factor cannot constitute a necessary condition for the unsafe behavior of high and non-high construction workers.
(2) Through the configuration perspective and fsQCA method, it is found that the unsafe behavior mechanism of tall construction workers is divided into five paths, which are summarized into three unsafe behavior driving modes. The first is the attitude-climate scarcity type which takes the absence of safety attitude and safety motivation as the core conditions. The second is psychological cognition scarcity type which Causal conditions are absent at the psychological cognition level. The third is the institutional environment scarcity type which Causal conditions are absent at the institutional environment level. The three types of unsafe behavior of construction workers differ in the causes of unsafe behavior. It reflects the differentiated matching between construction workers with different psychological safety cognition and various elements of the institutional environment and reflects the multiple realization methods of construction workers’ unsafe behavior. Therefore, according to the institutional environment of the project and the status quo of construction workers’ psychological cognition, each project manager can compare the five paths to realize the unsafe behavior of tall construction workers, which have similar paths. In the direction of attitude-climate dominance, psychological cognition dominance, and institutional environment dominance, the relationship between the safety cognition of construction workers and the environmental system should be properly handled to avoid the occurrence of unsafe behaviors of high-level construction workers. In addition, there is only one driving path for unsafe behavior of non-tall construction workers. Taking safety attitude, safety tools, system training, and safety climate as the core conditions, the “comprehensive type” assisted by institution control found an asymmetric relationship with the driving mechanism of unsafe behavior of tall construction workers. That is, the unsafe behavior path of tall construction workers is not the reverse path of the unsafe behavior of non-tall construction workers. According to the comprehensive type, project managers need to carry out comprehensive reforms to improve safety performance in all aspects.
(3) Among the five configurations of unsafe behaviors of tall construction workers, the configuration aspect: Compared with the psychological cognition scarcity type and the institutional environment scarcity type, the attitude-climate scarcity type is more likely to cause unsafe behavior of tall construction workers. Causal conditions: safety attitude (4 times) and safety climate (3 times) exist as core conditions. It shows that in the construction site, the lack of safety attitude and safety climate plays a key role in the production of unsafe behaviors.

### Theoretical Contribution

(1) This study integrates the theoretical framework of social cognition and examines the unsafe behavior mechanism of construction workers from five important Causal conditions at the level of psychological cognition and institutional environment. Previous studies are limited to the institutional environment or psychological cognition level, and the internal mechanism of the coupling and linkage of the two levels affecting the unsafe behavior of construction workers is still unclear. Therefore, an in-depth analysis of the synergistic linkage mechanism between the macro-institutional environment and the micro-psychological cognition level is carried out, and five paths affecting unsafe behaviors are found. It is also found that the path that is most likely to lead to unsafe behavior is helpful to unravel the black box of the institutional environment affecting unsafe behavior and to clarify the influence mechanism of institutional logic and micro-individuals. At the same time, since the unsafe behavior of construction workers is a complex problem, avoiding unsafe behavior is not determined by a single influencing factor, but depends on the different configurations of factors at the two levels of psychological cognition and institutional environment. For example, when explaining the inconsistency in the conclusion that the psychological cognitive level affects the unsafe behavior of construction workers, it may also be necessary to consider the elements of the institutional environment or the matching scenarios of elements at other levels. This improves the dilemma that previous studies ignore the influence of multiple factors on unsafe behavior, resulting in inconsistent research results.
(2) This study uses the fsQCA method to find that there is causal asymmetry in the unsafe behavior mechanism of construction workers. The paths that lead to high unsafe behavior are different from the paths that lead to non-high unsafe behavior, that is, the occurrence of unsafe behavior cannot be avoided according to the “traditional antithesis” of non-high unsafe behavior. At the same time, it is also found that there is a substitution relationship between the various elements in the configuration that affect the unsafe behavior of construction workers. For high insecurity behaviors, there are psychological cognition scarcity type, institutional environment scarcity type, and attitude-climate scarcity type. Under the condition of lack of attitude and climate, even if there is good safety training and good safety motivation, the lack of safety climate and safety motivation will play a substitute role to cause unsafe behavior. This fully reflects the advantages of the fsQCA method in explaining complex management issues, breaks through the limitations of traditional statistical methods, and enriches the research on safety behavior in management.
(3) Through the fsQCA method, six paths are found that lead to the unsafe behavior of tall and non-high construction workers, and the direction for construction enterprises to establish the goal of matching construction workers with the institutional environment is pointed out. Based on the framework of social cognition theory, an integrated analysis framework including psychological cognition and institutional environment factors is proposed, which enriches the application scope of social cognition theory.

### Management Implications

The findings of this study provide companies with effective strategies to avoid unsafe behavior among construction workers. Five types of drivers of high insecurity behaviors and one realization path of non-highly insecure behaviors are found in the study. It points out the direction for construction workers and enterprises to establish a match between construction workers and the institutional environment. First of all, for individuals who lack relevant attributes, such as safety attitude and safety climate among construction workers, in the construction stage, project managers should actively help construction workers to eliminate unsafe attitudes and carry out safety education and training within the project team through internal and external cooperation. At the same time, strengthen the safety responsibilities of relevant leaders, pay attention to safety, and establish a good organizational safety atmosphere. At the same time, this study shows that this combination has a large population, and safety precautions should be given priority.

Secondly, project managers lack psychological cognition, such people are under perfect conditions created by institution control, safety training, and safety atmosphere. However, when analyzing the personality differences of construction workers’ safety attitudes and safety motivations, we should try to intervene with different strategies. For example, assign reasonable work tasks, strengthen the humanized management of construction safety management, and publicize the safety of construction workers’ families, so as to improve the influence of family instructions on the safety attitude of construction workers. Ensure that the construction projects they are engaged in have a high level of safety attitude, and give corresponding support as much as possible to ensure that they continue to maintain this high safety attitude. Different strategies should be adopted for the institutional environment scarcity type. Managers will establish standardized and institutionalized safety education and technical disclosure for all construction workers. Regularly carry out safety training and assessment to improve the skill requirements for workers. Regular safety exchange meetings are held to promote exchanges between managers and team members, thereby promoting the improvement of technology, experience, and safety precautions, and ensuring the intensity and continuity of safety education and training. Therefore, project managers are required to track and intervene in the above external factors when necessary. Through communication between team members and management personnel, material rewards and punishments, safety education, and family notification are provided to construction workers to influence the safety attitude of construction workers from the outside to the mainland.

Finally, managers should actively build a corporate safety culture. Creating a positive safety culture is the embodiment of effective safety communication among team members, and indirectly affects the safety climate of the team, corrects the unsafe attitude and motivation of construction workers, and makes safety behavior a normal state in construction projects. It lays a solid foundation for enterprises to improve safety performance and gain competitive advantage.

### Limitations and Future Prospects

This study also has some limitations, which need to be further improved in future research: ①The 164 survey data in this study weaken the generalizability of the conclusions to a certain extent. In future research, more interview data of regional projects can be investigated to improve the universality of the conclusion. ② This study focuses on the influence of two factors at the psychological cognition and environmental level on the unsafe behavior of construction workers. Psychological factors are an important prerequisite for behavioral logic, but there are other factors that influence behavior. Therefore, future research can incorporate regional factors demographic characteristics, family factors, fatigue construction, and other factors that may affect construction workers’ unsafe conditions into the model to further improve case coverage. ③This study focuses on the construction workers’ safety perception and institutional environment, emphasizing the static, if the construction workers’ safety perception and institutional environment are changing, how to change the unsafe behavior of construction workers. Therefore, in the future, attempts can be made to collect dynamic data and further analyze the dynamic evolution of unsafe behaviors of construction workers.

## Data Availability Statement

The original contributions presented in the study are included in the article/supplementary material, further inquiries can be directed to the corresponding author/s.

## Ethics Statement

Written informed consent was obtained from the individual(s) for the publication of any potentially identifiable images or data included in this article.

## Author Contributions

BY: determine the topic selection, put forward the overall research ideas and framework of the paper, and write the first draft of the paper. SX and MN: paper modify. LC: data analysis and processing. All authors contributed to the article and approved the submitted version.

## Funding

This work was supported by Jiangxi Province Graduate Innovation Project: Study on the evaluation of residents’ satisfaction degree and its improvement path in the reconstruction of old residential areas (ID: YC2021-S592). Jiangxi Province Humanities and Social Science Project: Evaluation and countermeasures of old residential district reconstruction from the perspective of multiple subjects (ID: GL21119).

## Conflict of Interest

The authors declare that the research was conducted in the absence of any commercial or financial relationships that could be construed as a potential conflict of interest.

## Publisher’s Note

All claims expressed in this article are solely those of the authors and do not necessarily represent those of their affiliated organizations, or those of the publisher, the editors and the reviewers. Any product that may be evaluated in this article, or claim that may be made by its manufacturer, is not guaranteed or endorsed by the publisher.

## References

[ref1] Ajzen I. (1991). The theory of planned behavior. Organ. Behav. Hum. Decis. Process. 50, 179–211. doi: 10.1016/0749-5978(91)90020-T, PMID: 35303595

[ref2] Alizadeh S. S. Mortazavi S. B. Sepehri M. M.. (2015). Assessment of accident severity in the construction industry using the Bayesian theorem. Int. J. Occup. Saf. Ergon. 21, 551–557. doi: 10.1080/10803548.2015.1095546, PMID: 26694008

[ref3] Al-Refaie A. (2013). Factors affect companies’ safety performance in Jordan using structural equation modeling. Saf. Sci. 57, 169–178. doi: 10.1016/j.ssci.2013.02.010

[ref4] Alruqi W. M. Hallowell M. R. Ulises T. (2018). Safety climate dimensions and their relationship to construction safety performance: a meta-analytic review. Saf. Sci. 109, 165–173. doi: 10.1016/j.ssci.2018.05.019

[ref5] Amponsah-Tawaih K. Appiah M. A. (2016). Work pressure and safety behaviors among health workers in Ghana: the moderating role of management commitment to safety. Safety Health Work 7, 340–346. doi: 10.1016/j.shaw.2016.05.001, PMID: 27924238 PMC5128006

[ref6] Andriessen J. (1978). Safe behaviour and safety motivation. J. Occup. Accid. 1, 363–376. doi: 10.1016/0376-6349(78)90006-8, PMID: 35065980

[ref7] Austin J. Kessler K. L. Riccobono J. E.. (1996). Using feedback and reinforcement to improve the performance and safety of a roofing crew. J. Organ. Behav. Manag. 16, 49–75. doi: 10.1300/J075v16n02_04

[ref8] Bandura A. (2001). Social cognitive theory: an agentive perspective. Annu. Rev. Psychol. 52, 1–26. doi: 10.1146/annurev.psych.52.1.1, PMID: 11148297

[ref001] Bandura A. Cervone D. (1986). Differential engagement of self-reactive influences in cognitive motivation. Organ. Behav. Hum. Decis. Process. 38, 92–113. doi: 10.1016/0749-5978(86)90028-2

[ref9] Brondino M. Silva S. A. Pasini M. (2012). Multilevel approach to organizational and group safety climate and safety performance: co-workers as the missing link. Saf. Sci. 50, 1847–1856. doi: 10.1016/j.ssci.2012.04.010

[ref10] Burns C. Conchie S. (2014). Risk information source preferences in construction workers. Empl. Relat. 36, 70–81. doi: 10.1108/er-06-2013-0060

[ref11] Busenitz L. W. Gómez C. Spencer J. W. (2000). Country institutional profiles: unlocking entrepreneurial phenomena. Acad. Manag. J. 43, 994–1003. doi: 10.2307/1556423

[ref13] Chang J. H. Han S. Abou Rizk S. M. Kanerva J. (2019). Stratified statistical analysis for effectiveness evaluation of frontline worker safety intervention: case study of construction steel fabrication. Saf. Sci. 115, 89–102. doi: 10.1016/j.ssci.2019.01.030

[ref14] Chang S. J. Koh S. B. Kang D. Kim S. A. Kang M. G. Lee C. G.. (2005). Developing an occupational stress scale for Korean employees. Kor. J. Occup. Environ. Med. 17, 297–317. doi: 10.35371/kjoem.2005.17.4.297, PMID: 31410868

[ref15] Chang Y. H. J. Mosleh A. (2007). Cognitive modeling and dynamic probabilistic simulation of operating crew response to complex system accidents. Part 5: dynamic probabilistic simulation of the IDAC model. Reliab. Eng. Syst. Saf. 92, 1076–1101. doi: 10.1016/j.ress.2006.05.012

[ref16] Chen M. Chen C. C. Sheldon O. J. (2016). Relaxing moral reasoning to win: how organizational identification relates to unethical pro-organizational behavior. J. Appl. Psychol. 101, 1082–1096. doi: 10.1037/apl0000111, PMID: 27100068

[ref002] Cheng B. Fan C. Fu H. Huang J. Chen H. Luo X. (2022). Measuring and computing cognitive statuses of construction workers based on electroencephalogram: a critical review. IEEE Trans. Comput. Soc. Syst. doi: 10.1109/TCSS.2022.3158585 [Epub ahead of print].

[ref17] Chen Y. McCabe B. Hyatt D. (2017). Impact of individual resilience and safety climate on safety performance and psychological stress of construction workers: a case study of the Ontario construction industry. J. Saf. Res. 61, 167–176. doi: 10.1016/j.jsr.2017.02.014, PMID: 28454862

[ref18] Cheyne A. Cox S. Oliver A. Tomás J. M. (1998). Modelling safety climate in the prediction of levels of safety activity. Work Stress 12, 255–271. doi: 10.1080/02678379808256865, PMID: 23708470

[ref19] Choi B. Lee S. (2018). An empirically based agent-based model of the sociocognitive process of construction workers’ safety behavior. J. Constr. Eng. Manag. 144:04017102. doi: 10.1061/(ASCE)CO.1943-7862.0001421

[ref20] Choudhry RM. (2008). Exploratory Study of the Safety Culture in Construction. Beijing: Tsinghua University.

[ref21] Choudhry R. M. (2014). Behavior-based safety on construction sites: a case study. Accid. Anal. Prev. 70, 14–23. doi: 10.1016/j.aap.2014.03.007, PMID: 24686162

[ref22] Choudhry R. M. Fang D. (2008). Why operatives engage in unsafe work behavior: investigating factors on construction sites. Saf. Sci. 46, 566–584. doi: 10.1016/j.ssci.2007.06.027

[ref23] Conchie S. M. (2013). Transformational leadership, intrinsic motivation, and trust: a moderated-mediated model of workplace safety. J. Occup. Health Psychol. 18, 198–210. doi: 10.1037/a0031805, PMID: 23506550

[ref25] Dekker S. W. (2002). Reconstructing human contributions to accidents: the new view on error and performance. J. Saf. Res. 33, 371–385. doi: 10.1016/S0022-4375(02)00032-4, PMID: 12404999

[ref26] Ding L. Fang W. Luo H. Love P. E. Zhong B. Ouyang X. (2018). A deep hybrid learning model to detect unsafe behavior: integrating convolution neural networks and long short-term memory. Autom. Constr. 86, 118–124. doi: 10.1016/j.autcon.2017.11.002

[ref27] Du Y. Kim P. H. (2021). One size does not fit all: strategy configurations, complex environments, and new venture performance in emerging economies - sciencedirect. J. Bus. Res. 124, 272–285. doi: 10.1016/j.jbusres.2020.11.059, PMID: 35305451

[ref28] Duma K. Husodo A. H. Soebijanto Maurits L. S. (2014). The policy of control health and safety and the risk factors in the coal mining of East Kalimantan. BMC Public Health 14:1. doi: 10.1186/1471-2458-14-S1-O2624383435

[ref29] Eagly A. H. Chaiken S. (1993). The psychology of attitudes, Harcourt brace jovanovich college publishers. J. Loss Prev. Process Ind. 8, 299–305.

[ref30] Edwards J. R. (2008). 4 person-environment fit in organizations: an assessment of theoretical progress. Acad. Manag. Ann. 2, 167–230. doi: 10.5465/19416520802211503

[ref31] Fam I. M. Ghasemi F.. (2017). Constructing a Bayesian network model for im-proving safety behavior of employees at workplaces. Appl. Ergon. 58, 35–47. doi: 10.1016/j.apergo.2016.05.006 27633196

[ref32] Fang D. P. (2006). Safety climate in construction industry: a case study in Hong Kong. J. Construct. Eng. Manage. 132, 573–584. doi: 10.1061/(ASCE)0733-9364(2006)132:6(573)

[ref33] Fang W. Ding L. Luo H. Love P. E. (2018). Falls from heights: a computer vision-based approach for safety harness detection. Autom. Constr. 91, 53–61. doi: 10.1016/j.autcon.2018.02.018

[ref34] Fang D. Jiang Z. Zhang M. Wang H. (2015). An experimental method to study the effect of fatigue on construction workers’ safety performance. Saf. Sci. 73, 80–91. doi: 10.1016/j.ssci.2014.11.019

[ref36] Fang D. Zhao C. Zhang M. (2016). A cognitive model of construction workers’ unsafe behaviors. J. Constr. Eng. Manag. 142:04016039. doi: 10.1061/(ASCE)CO.1943-7862.0001118, PMID: 34975693

[ref37] Fargnoli M. Lombardi M. (2019). Preliminary human safety assessment (PHSA) for the improvement of the behavioral aspects of safety climate in the construction industry. Buildings 9:69. doi: 10.3390/buildings9030069

[ref38] Feng Y. Zhang S. Wu P. (2015). Factors influencing workplace accident costs of building projects. Saf. Sci. 72, 97–104. doi: 10.1016/j.ssci.2014.08.008

[ref39] Fiss P. C. (2007). A set-theoretic approach to organizational configurations. Acad. Manag. Rev. 32, 1180–1198. doi: 10.5465/amr.2007.26586092

[ref40] Fiss P. C. (2011). Building better causal theories: a fuzzy set approach to typologies in organization research. Acad. Manag. J. 54, 393–420. doi: 10.5465/amj.2011.60263120

[ref41] Fornell C. Larcker D. F. (1981). Evaluating structural equation models with unobservable variables and measurement error. J. Mark. Res. 18, 39–50. doi: 10.1177/002224378101800104

[ref43] Fu G. Xie X. Jia Q. Li Z. Chen P. Ge Y. (2020). The development history of accident causation models in the past 100 years: 24Model, a more modern accident causation model - ScienceDirect. Process Saf. Environ. Prot. 134, 47–82. doi: 10.1016/j.psep.2019.11.027

[ref44] Goh Y. M. Binte Sa’adon N. F. (2015). Cognitive factors influencing safety behavior at height: a multimethod exploratory study. J. Constr. Eng. Manag. 141:04015003. doi: 10.1061/(ASCE)CO.1943-7862.0000972

[ref45] Greckhamer T. (2016). CEO compensation in relation to worker compensation across countries: the configurational impact of country-level institutions. Strateg. Manag. J. 37, 793–815. doi: 10.1002/smj.2370

[ref46] Greckhamer T. Furnari S. Fiss P. C. Aguilera R. V. (2018). Studying configurations with qualitative comparative analysis: best practices in strategy and organization research. Strateg. Organ. 16, 482–495. doi: 10.1177/1476127018786487

[ref47] Griffin M. A. Neal A. (2000). Perceptions of safety at work: a framework for linking safety climate to safety performance, knowledge, and motivation. J. Occup. Health Psychol. 5, 347–358. doi: 10.1037/1076-8998.5.3.347, PMID: 10912498

[ref003] Guo X. Liu X. Chen S. Li L. Fu H. (2021). China’s housing provision system: evolution, purchase -rental gap measurement and optimization strategy. J. Urban Plann. Dev. 147:04021054.

[ref48] Guo B. Yiu T. W. González V. A. (2016a). Predicting safety behavior in the construction industry: development and test of an integrative model – sciencedirect. Saf. Sci. 84, 1–11. doi: 10.1016/j.ssci.2015.11.020

[ref49] Guo B. H. Yiu T. W. González V. A. (2016b). Predicting safety behavior in the construction industry: development and test of an integrative model. Saf. Sci. 84, 1–11. doi: 10.1016/j.ssci.2015.11.020

[ref50] Guo B. Zhang L. Li Y. (2019). Research on the path of residents’ willingness to upgrade by installing elevators in old residential quarters based on safety precautions. Saf. Sci. 118, 389–396. doi: 10.1016/j.ssci.2019.05.038

[ref53] Hair J. F. Ringle C. M. Sarstedt M. (2011). PLS-SEM: indeed a silver bullet. J. Mark. Theory Pract. 19, 139–152. doi: 10.2753/MTP1069-6679190202

[ref54] Hasanzadeh S. Esmaeili B. Dodd M. D. (2018). “Examining the relationship between personality characteristics and worker’s attention under fall and tripping hazard conditions.” in *Safety and Disaster Management, Proceedings of the Construction Research Congress 2018*, New Orleans, LA, USA, 2–4 April 2018; ASCE: Reston, VA, USA, pp. 412–422.

[ref55] Heinrich H. S. W., (1931). Industrial Accident Prevention: A Scientific Approach. New York: McGraw-Hill Book Company, Incorporated.

[ref56] Henning J. B. Stufft C. J. Payne S. C. Bergman M. E. Mannan M. S. Keren N. (2009). The influence of individual differences on organizational safety attitudes. Saf. Sci. 47, 337–345. doi: 10.1016/j.ssci.2008.05.003, PMID: 33829528

[ref57] Higgins C. C. A. (1995). Computer self-efficacy: development of a measure and initial test. MIS Q. 19, 189–211. doi: 10.2307/249688

[ref59] Iyer P. S. Haight J. M. Del Castillo E. Tink B. W. Hawkins P. W. (2004). Intervention effectiveness research: understanding andoptimizing industrial safety programs using leading indicators. Chem. Health Saf. 11, 9–19. doi: 10.1016/j.chs.2003.10.003

[ref61] Jiang Z. Fang D. Zhang M. (2015). Understanding the causation of construction workers unsafe behaviors based on system dynamics modeling. Am. Soc. Civil Eng. 31:04014099. doi: 10.1061/(ASCE)ME.1943-5479.0000350

[ref62] Jiang L. Tetrick L. E. (2016). Mapping the nomological network of employee self-determined safety motivation: a preliminary measure in China. Accid. Anal. Prev. 94, 1–7. doi: 10.1016/j.aap.2016.05.009, PMID: 27240123

[ref63] Jitwasinkul B. Hadikusumo B. H. W. Memon A. Q. (2016). A Bayesian belief network model oforganizational factors for improving safe work behaviors in Thai construction in-dustry. Saf. Sci. 82, 264–273. doi: 10.1016/j.ssci.2015.09.027

[ref64] Jokkaw N. Suteecharuwat P. Weerawetwat P. (2017). Measurement of construction workers’ feeling by virtual environment (ve) technology for guardrail design in high-rise building construction projects. Eng. J. 21, 161–177. doi: 10.4186/ej.2017.21.5.161

[ref65] Ju D. Qin X. Xu M. DiRenzo M. S. (2016). Boundary conditions of the emotional exhaustion-unsafe behavior link: the dark side of group norms and personal control. Asia Pac. J. Manag. 33, 113–140. doi: 10.1007/s10490-015-9455-7

[ref66] Judge W. Q. Fainshmidt S. Brown J. L. (2020). Institutional systems for equitable wealth creation: replication and an up⁃ date of judge et al. (2014). Manag. Organ. Rev. 16, 5–31. doi: 10.1017/mor.2020.1

[ref67] Khosravi Y. Asilian-Mahabadi H. Hajizadeh E. Hassanzadeh-Rangi N. Bastani H. Khavanin A.. (2014). Modeling the factors affecting unsafe behavior in the construction industry from safety supervisors’ perspective. J. Res. Health Sci. 14, 29–35. PMID: 24402847

[ref68] Kim J. Lee H. Park M. Kwon N. (2017). A system dynamics approach for modeling cognitive process of construction workers' unsafe behaviors. Korean J. Constr. Eng. Manage. 18, 38–48. doi: 10.6106/KJCEM.2017.18.2.038, PMID: 24268437

[ref004] Kim K. W. Lim H. C. Park J. H. Park S. G. Park Y. J. Cho H. H. (2018). Developing a basic scale for workers’ psychological burden from the perspective of occupational safety and health. Saf. Health Work. 224–231. doi: 10.1016/j.shaw.2018.02.004 29928538 PMC6005905

[ref69] Kim N. K. Rahim N. Iranmanesh M. Foroughi B. (2019). The role of the safety climate in the successful implementation of safety management systems. Saf. Sci. 118, 48–56. doi: 10.1016/j.ssci.2019.05.008

[ref70] Koestner R. Hope N. (2014). “A self-determination theory approach to goals,” in Oxford Handbook of Work Engagement, Motivation, and Self Determination Theory. ed. Gagné M. (New York: Oxford University Press)

[ref71] Langford D. Rowlinson S. Sawacha E. (2000). Safety behavior and safety management: itsinfluence on the attitudes of workers in the UK construction industry. Eng. Construct. Archit. Manage. 7, 133–140. doi: 10.1108/eb021138

[ref72] Lewin K. (1976). Field Theory in Social Science: Selected Theoretical Papers. Chicago, IL: University of Chicago Press.

[ref73] Li H. Li X. Luo X. Siebert J. (2017). Investigation of the causality patterns of non-helmet us behavior of construction workers. Autom. Constr. 80, 95–103. doi: 10.1016/j.autcon.2017.02.006

[ref74] Li H. Lu M. J. Hsu S. C. Gray M. Huang T. (2015). Proactive behavior-based safety management for construction safety improvement. Saf. Sci. 75, 107–117. doi: 10.1016/j.ssci.2015.01.013

[ref75] Liang H. Saraf N. Xue H. Y. (2007). Assimilation of enterprise systems: the effect of institutional pressures and the mediating role of top management. MIS Q. 31, 59–87. doi: 10.2307/25148781

[ref76] Liao P. C. Lei G. P.. (2014). The relationship between communication and con- struction safety climate in China. KSCE J. Civ. Eng. 18, 887–897. doi: 10.1007/s12205-014-0492-4

[ref78] Llopis-Albert C. Rubio F. Valero F. (2019). Fuzzy-set qualitative comparative analysis applied to the design of a network flow of automated guided vehicles for improving business productivity. J. Bus. Res. 101, 737–742. doi: 10.1016/j.jbusres.2018.12.076

[ref79] Man S. S. Chan A. H. S. Alabdulkarim S. (2019). Quantification of risk perception: development and validation of the constructionworker risk perception (CoWoRP) scale. J. Saf. Res. 71, 25–39. doi: 10.1016/j.jsr.2019.09.009, PMID: 31862036

[ref81] March J. G. (2010). The Ambiguities of Experience. New York, US: Cornell University Press.

[ref82] Martínez-Córcoles M. Stephanou K. (2017). Linking active transactional leadership and safety performance in military operations. Saf. Sci. 96, 93–101. doi: 10.1016/j.ssci.2017.03.013

[ref83] Meng Q. Liu W. Li Z. Hu X. (2021). Influencing factors, mechanism and prevention of construction workers’ unsafe behaviors: a systematic literature review. Int. J. Environ. Res. Public Health 18:2644. doi: 10.3390/ijerph18052644, PMID: 33807980 PMC7967310

[ref84] Meyer A. D. Tsui A. S. Hinings C. R. (1993). Configurational approaches to organizational analysis. Acad. Manag. J. 36, 1175–1195.

[ref85] Mohajeri M. Ardeshir A. Malekitabar H. Rowlinson S. (2021). Structural model of internal factors influencing the safety behavior of construction workers. J. Constr. Eng. Manag. 147:04021156. doi: 10.1061/(ASCE)CO.1943-7862.0002182, PMID: 26775077

[ref86] Mohamed S. (2002). Safety climate in construction site environments. J. Construct. Eng. Manage. 128, 375–384. doi: 10.1061/(ASCE)0733-9364(2002)128:5(375), PMID: 29448922

[ref88] Neal A. Griffin M. A. (2004). “Safety climate and safety at work,” in The Psychology of Work Place Safety. eds. Barling J. Frone M. (Washington, DC: American Psychological Association), 15–34.

[ref89] Neal A. Griffin M. Hart P. (2000). The impact of organizational climate on safety climate and individual behavior. Saf. Sci. 34, 99–109. doi: 10.1016/S0925-7535(00)00008-4, PMID: 34704531

[ref90] Nicole D. François B. (1991). A safety climate measure for construction sites. J. Saf. Res. 22, 97–103.

[ref91] NIOSH (National Institute of Occupational Safety and Health) (2015).“Fatality Assessment and Control (FACE) program.” Available at: http://www.cdc.gov/niosh/face/default.html〉 (November 15, 2016).

[ref92] Panuwatwanich K. Al-Haadir S. Stewart R. A. (2016). Influence of safety motivation and climate on safety behaviour and outcomes: evidence from the saudiarabian construction industry. Int. J. Occup. Safety Ergon. Jose 23, 60–75. doi: 10.1080/10803548.2016.1235424, PMID: 27617673

[ref93] Panuwatwanich K. Al-Haadir S. Stewart R. A. (2017). Influence of safety motivation and climate on safety behaviour and outcomes: evidence from the Saudi Arabian construction industry. Int. J. Occup. Saf. Ergon. 23, 60–75. doi: 10.1080/10803548.2016.1235424. PMID: 27617673, PMID: 27617673

[ref94] Pappas I. O. Kourouthanassis P. E. Giannakos M. N. Chrissikopoulos V. (2016). Explaining online shopping behavior with fsQCA: the role of cognitive and affective perceptions. J. Bus. Res. 69, 794–803. doi: 10.1016/j.jbusres.2015.07.010

[ref95] Park Y. K. Mithas S. (2020). Organized complexity of digital business strategy: a configurational perspective. MIS Q. 44, 85–127. doi: 10.25300/MISQ/2020/14477

[ref96] Petersen D. (1971). Techniques of Safety Management. McGraw-Hill: New York, NY, USA.

[ref97] Ragin C. C. (2008). Redesigning Social Inquiry: Fuzzy Sets and beyond. Vol. 240. Hoboken, NJ: Wiley Online Library.

[ref98] Reason J. (1995). A systems approach to organizational error. Ergonomics 38, 1708–1721. doi: 10.1080/00140139508925221, PMID: 35271020

[ref99] Rigby C. S. Ryan R. M. (2018). Self-determination theory in human resource development: new directions and practical considerations. Adv. Dev. Hum. Resour. 20, 133–147. doi: 10.1177/1523422318756954

[ref100] Rihoux B. Ragin C. C. (2008). Configurational Comparative Methods: Qualitative Comparative Analysis (QCA) and Related Techniques. Thousand Oaks, CA: Sage Publications.

[ref101] Rihoux B. Ragin C. C. (2009). Configurational Comparative Methods: Qualitative Comparative Analysis (QCA) and Related Techniques. Vol. 51. Thousand Oaks, CA: Sage Publications.

[ref102] Rundmo T. Hale A. R. (2003). Managers’ attitudes toward safety and accident prevention. Saf. Sci. 41, 557–574. doi: 10.1016/S0925-7535(01)00091-1, PMID: 23991508

[ref103] Ryan R. M. Deci E. L. (2008). From ego depletion to vitality: theory and findings concerning the facilitation of energy available to the self. Soc. Personal. Psychol. Compass 2, 702–717. doi: 10.1111/j.1751-9004.2008.00098.x

[ref105] Sampson J. M. DeArmond S. Chen P. Y. (2014). Role of safety stressors and social support on safety performance. Saf. Sci. 64, 137–145. doi: 10.1016/j.ssci.2013.11.025, PMID: 31635232

[ref106] Schneider C. Q. Wagemann C. (2012). Set-theoretic methods for the social sciences: a guide to qualitative comparative analysis. doi: 10.1017/CBO9781139004244.

[ref108] Scott N. Fleming M. Kelloway E. K. (2014). “Understanding why employees behave safely from a self-determination theory perspective,” in Oxford Handbook of Work Engagement, Motivation, and Self-Determination Theory. eds. Gagne M. Nathan P. E. (New York: Oxford University Press), 276–294.

[ref109] Shea T. Cieri H. D. Vu T. Pettit T. (2021). How is safety climate measured? A review and evaluation. Saf. Sci. 143:105413. doi: 10.1016/j.ssci.2021.105413

[ref110] Shen Y. Tuuli M. M. Xia B. Koh T. Y. Rowlinson S. (2015). Toward a model for forming psychological safety climate in construction project management. Int. J. Proj. Manag. 33, 223–235. doi: 10.1016/j.ijproman.2014.04.009

[ref111] Shin M. Lee H. S. Park M. Moon M. Han S. (2014). A system dynamics approach for modeling construction workers’ safety attitudes and behaviors. Accid. Anal. Prev. 68, 95–105. doi: 10.1016/j.aap.2013.09.019, PMID: 24268437

[ref113] Suto M. (2009). Safety reserch intitute of JR west. Jap. Railway Eng. 18–19.

[ref114] Tett R. P. Guterman H. A. (2000). Situation trait relevance, trait expression, and cross-situational consistency: testing a principle of trait activation. J. Res. Pers. 34, 397–423. doi: 10.1006/jrpe.2000.2292

[ref115] Thomas G. Santi F. Fiss P. C. Aguilera R. V. (2018). Studying configurations with qualitative comparative analysis: best practices in strategy and organization research. Strateg. Organ. 147612701878648. doi: 10.1177/1476127018786487

[ref116] Toole T. M. (2002). Construction site safety roles. J. Constr. Eng. Manag. 128, 203–210. doi: 10.1061/(ASCE)0733-9364(2002)128:3(203), PMID: 35206384

[ref117] Ulleberg P. Rundmo T. (2003). Personality, attitudes and risk perception as predictors of risky driving behaviour among young drivers. Saf. Sci. 41, 427–443. doi: 10.1016/S0925-7535(01)00077-7

[ref118] Weidman J. Dickerson D. E. Koebel C. T. (2015). Intervention to improve purchasing decision-maker perceptions of ventilated tools. J. Constr. Eng. Manag. 141:04015007. doi: 10.1061/(ASCE)CO.1943-7862.0000961,04015007

[ref119] Wu J. Li Y. Zhang D. (2019). Identifying women’s entrepreneurial barriers and empowering female entrepreneurship worldwide: a fuzzy-set qca approach. Int. Entrep. Manag. J. 15, 905–928. doi: 10.1007/s11365-019-00570-z

[ref120] Xu S. Zou P. X. W. Luo H. B. (2018). Impact of attitudinal ambivalence on safety behaviour in construction. Adv. Civ. Eng. 2018, 1–12. doi: 10.1155/2018/7138930

[ref121] Yang Y. K. Byung-Seok K. (2014). Study on the structural relation between the level of fatigue and stress of construction workers and disaster risks. J. Korea. Saf. Manag. Sci. 16, 35–44. doi: 10.12812/ksms.2014.16.3.35

[ref122] Yang J. Ye G. Xiang Q. Kim M. Yue H. (2021). Insights into the mechanism of construction workers’ unsafe behaviors from an individual perspective. Saf. Sci. 133:105004. doi: 10.1016/j.ssci.2020.105004

[ref123] Zaira M. M. Hadikusumoa B. H. W. (2017). Structural equation model of integrated safety intervention practices affecting the safety behaviour of workers in the construction industry. Saf. Sci. 98, 124–135. doi: 10.1016/j.ssci.2017.06.007

[ref124] Zamani V. (2020). Seyed Yaser Banihashemi, Alireza Abbasi, how can communication networks among excavator crew members in construction projects affect the relationship between safety climate and safety outcomes? Saf. Sci. 128:104737. doi: 10.1016/j.ssci.2020.104737

[ref125] Zhang J. S. Fu J. Hao H. Y. Fu G. Nie F. C. Zhang W. Y. (2020a). Root causes of coal mine accidents: characteristics of safety culture deficiencies based on accident statistics – sciencedirect. Process Saf. Environ. Prot. 136, 78–91. doi: 10.1016/j.psep.2020.01.024

[ref127] Zhang P. Li N. Jiang Z. Fang D. Anumba C. J. (2019). An agent-based modeling approach for understanding the effect of worker-management interactions on construction workers’ safety-related behaviors. Autom. Constr. 97, 29–43. doi: 10.1016/j.autcon.2018.10.015

[ref128] Zhang J. Xiang P. C. Zhang R. Chen D. Ren Y. T. (2020b). Mediating effect of risk propensity between personality traits and unsafe behavioral intention of construction workers. J. Constr. Eng. Manag. 146:04020023. doi: 10.1061/(ASCE)CO.1943-7862.0001792

[ref129] Zhou Q. A. Fang D. P. Mohamed S. (2011). Safety climate improvement: case study in a Chinese construction company. J. Constr. Eng. Manag. Asce. 137, 86–95. doi: 10.1061/(ASCE)CO.1943-7862.0000241

[ref130] Zohar D. (1980). Safety climate in industrial organizations: theoretical and applied implications. J. Appl. Psychol. 65, 96–102. doi: 10.1037/0021-9010.65.1.96, PMID: 7364709

[ref131] Zohar D. Luria G. (2005). A multilevel model of safety climate: cross-level relationships between organization and group-level climates. J. Appl. Psychol. 90, 616–628. doi: 10.1037/0021-9010.90.4.616, PMID: 16060782

